# Hypopharyngeal Dedifferentiated Liposarcoma in the *MDM2* Era: A Case Report and Short Review

**DOI:** 10.1155/2020/2968467

**Published:** 2020-03-11

**Authors:** Peter Wanes, David A. Nolte, Ghassan A. Tranesh

**Affiliations:** Department of Pathology, University of Arizona, 1501 N. Campbell Ave, Tucson, AZ 85724, USA

## Abstract

Pharyngeal liposarcomas are very rare; still more rare are dedifferentiated liposarcomas in the pharynx. An 82-year-old man presented with dysphagia, voice changes, weight loss, nasal regurgitation of liquids, and coughing spells. A 3.5 cm mass was identified in the hypopharynx. The mass was biopsied and diagnosed as a benign fibroepithelial polyp. Continued symptoms and airway obstruction prompted a pharyngectomy, and the mass was then diagnosed as dedifferentiated liposarcoma. Due to infrequency and subtle histological findings, liposarcomas of the head and neck can be misdiagnosed and recur.

## 1. Introduction

Liposarcoma comprises only 1% of head and neck sarcomas [1]; and sarcomas are less than 1% of all neoplasms of the head and neck [2]. Thus, it is very rare—approximately 0.01% of head and neck tumors. In this region, liposarcoma most commonly occurs in the subcutaneous tissue (81%), with only 2.2% involving the larynx [3]. This tumor can dedifferentiate to a more aggressive form, as reported in the following case.

## 2. Case Report

### 2.1. Subject

**Figure 1:**
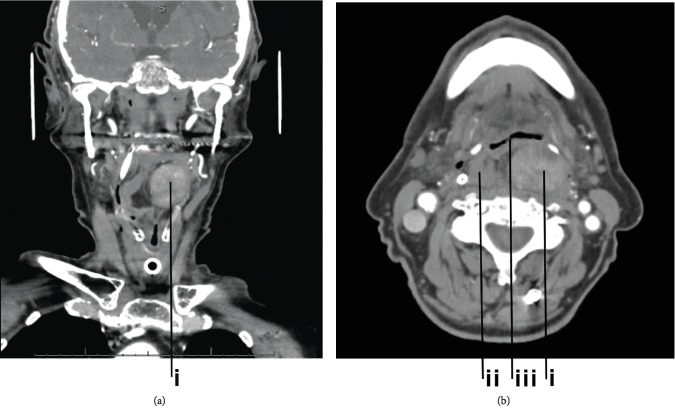
CT angiogram of neck ((a) coronal, (b) axial) demonstrating a 3.5 cm heterogeneous lesion in the posterior wall of the hypopharynx in a submucosal location (i) and a smaller 1.7 cm lesion of the right posterior wall of the hypopharynx (ii). The mass caused severe narrowing of the air column at the hypopharynx (iii).

An 82-year-old man presented to the clinic for evaluation of a throat mass. He reported dysphagia that began 4 to 5 months previously with associated 17-pound weight loss. He denied throat pain but endorsed voice changes, frequent nasal regurgitation of liquids, food getting stuck, and frequent coughing spells. After a prolonged coughing episode, he went to urgent care and was given an analgesic with symptomatic improvement. However, his dysphagia progressed until he developed difficulty swallowing water. He had no family history of cancer. He went to his primary care physician for evaluation who referred him to gastroenterology for an upper endoscopy and a Modified Barium Swallow Study. The upper endoscopy revealed no concerns with his esophagus, but a referral to otolaryngology was recommended. In the office, a flexible laryngoscopy revealed a benign-appearing mass in his throat. A CT scan of the neck (Figure 1) showed a 3.5 cm in diameter hyperdense mass in the hypopharynx originating in the left dorsal wall and a smaller 1.7 cm mass in the right posterior hypopharynx. A biopsy of the mass was diagnosed as a benign fibroepithelial polyp. Unfortunately, the patient continued to experience airway obstruction; thus, a transoral robot-assisted pharyngectomy was performed to remove this mass. The tissue was sent to pathology for further evaluation. The case was sent for expert consultation where it was diagnosed as dedifferentiated liposarcoma, intermediate grade (grade 2 of 3, Fédération Nationale des Centres de Lutte Contre le Cancer (FNCLCC)) arising from a background well-differentiated liposarcoma.

### 2.2. Gross Examination

The resected mass was four tan and soft, ovoid rubbery portions with focal overlying gray-pink glistening mucosa that measured 3.1-4.5 cm. Opposite the mucosal surfaces was an abundant cautery artefact. Cut sections showed pale tan to gray solid lobulated tissue; the largest with slightly edematous gray-white whorled cut surface that showed no degenerative changes.

### 2.3. Microscopic Examination

**Figure 2:**
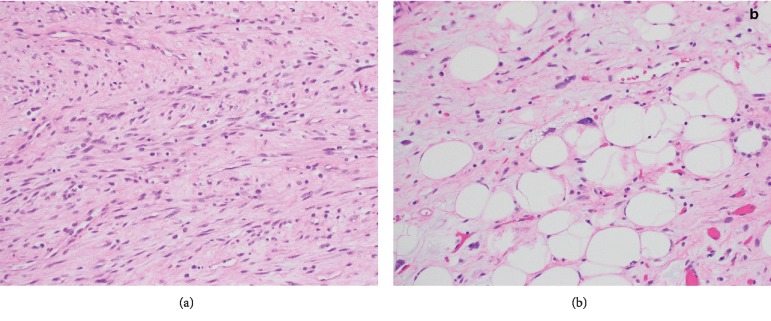
(a) Dedifferentiated liposarcoma, solid fascicles of spindle cells with eosinophilic cytoplasm and slightly enlarged nuclei; (b) well-differentiated area, adipocytes, and lipoblasts with enlarged, hyperchromatic nuclei and multivacuolated cytoplasm (hematoxylin and eosin, 200x magnification).

On microscopic examination, the neoplasm was predominantly composed of dedifferentiated areas with solid sheets of spindle cells in a fascicular and storiform pattern (Figure 2(a)). There was a minor component of well-differentiated soft tissue, from which the dedifferentiated liposarcoma likely arose, composed of adipocytes and atypical lipoblasts with enlarged, hyperchromatic nuclei with multivacuolated cytoplasm (Figure 2(b)). Focal myxoid changes were present, but no necrosis or atypical mitoses were observed. The cauterized margins appeared to be involved by both the well-differentiated and dedifferentiated tumor components.

### 2.4. Immunohistochemistry

To further characterize the mass, immunohistochemical stains were ordered with results as follows: focally positive for BCL2, while negative for CD99, S100, CK OSCAR, SOX10, SMA, desmin, CD31, CD34, and ERG. Beta catenin was noncontributory. This profile helped to rule out other tumor types.

### 2.5. Molecular Studies

To support the diagnosis, fluorescence in situ hybridization (FISH) was performed. The posterior pharyngeal mass demonstrated amplification of *MDM2* (12q15) by FISH, confirming the diagnosis of a liposarcoma.

### 2.6. Follow-Up

At clinical follow-up, approximately one month after pharyngectomy, the patient reported improvement and was able to eat and drink without problems. He denied dysphagia, dyspnea, and odynophagia and began to gain weight. Laryngoscopy revealed the surgical site to be well healed and no obstruction of the pharyngeal airway was seen. A full-body PET CT scan was negative for avidity in the pharynx or elsewhere. A neck CT scan showed a 2.2 cm thickening of the posterior pharynx with fat attenuation, possibly representing residual liposarcoma, as the margins of the pharyngectomy specimen were thought to be microscopically involved by this tumor. At multidisciplinary tumor board, it was decided that a repeat CT scan will be obtained in three months to reevaluate the laryngeal thickening. Further treatment decisions may be made at that time.

## 3. Discussion

Liposarcoma is a malignant neoplasm of adipose tissue and may recur locally but usually does not metastasize unless dedifferentiated [4]. Dedifferentiation occurs in approximately 10% of all liposarcomas [4]. In all sites including outside the head and neck, liposarcoma is the most common tumor among sarcomas of the soft tissue and is categorized into four subgroups: atypical lipomatous tumor/well-differentiated liposarcoma (WDLS) [5], dedifferentiated liposarcoma (DDLS), myxoid liposarcoma, and pleomorphic liposarcoma [4]. DDLS is an aggressive malignancy; hence, its recurrence and metastasis rates are higher than those of other types of liposarcoma [3, 6, 7].

Pharyngeal liposarcomas are very rare. Fewer than 100 have been reported in the literature. They predominantly (65%) occur in men [1]. Patients of all age groups are affected (6 months-86 years), with the median of patients in their 7th decade of life [1]. Clinical presentations usually include progressive difficulty breathing and swallowing of several weeks up to several years. The mass can be viewed by laryngoscopy as a polypoid, pedunculated mass. Long polyps may be sausage-like and regurgitatable. The clinical course is typically characterized by multiple recurrences, with intervals from several months, up to 26 years [8]. Dedifferentiation typically occurs late [9] and has been associated with distant metastasis and worse prognosis [10]. Location and histological grade may be the main determinants of prognosis with laryngeal tumors being better than oral tumors [9].

Symptoms vary by the site of involvement and tumor size. In the larynx, there is hoarseness, dysphonia, dysphagia, and airway obstruction. In the pharynx and larynx, there are signs of dysphagia and airway obstruction, respectively, accompanied by a slowly growing painless mass. The treatment of choice is wide local surgical excision that includes tumor-free margins [8]. More aggressive surgical procedures may be indicated for higher-grade histologic variants [8]. Adjuvant radiation treatment is controversial [8]. The clinical course is characterized by multiple local recurrences, often not recognized as liposarcoma. The 5-year survival rate for all liposarcomas of the head and neck is around 67% [8] and likely worse for dedifferentiated liposarcoma. The prognosis is better in the larynx compared to sites outside the head and neck (e.g., retroperitoneum), possibly due to earlier detection [9].

The differential diagnosis of a pharyngeal mass includes benign and malignant entities such as fibrovascular polyp, lipoma, nodular fasciitis, carcinoma, sarcoma, lymphoma, and melanoma. Discriminating liposarcoma from lipoma by histology alone can be difficult, as the cytologic atypia of a well-differentiated liposarcoma can be subtle. Indeed, the majority of hypopharyngeal/esophageal WDLS in the past have been considered reactive polyps rather than malignant neoplasms [10]. In addition, polypoid masses at this location are subject to constant trauma, which can cause secondary changes such as hemorrhage, calcification, fat necrosis, and infarction. Immunohistochemistry is not very useful, except to exclude alternate diagnoses.

Detecting *MDM2* or *CDK4* amplification by FISH is a sensitive and specific method for distinguishing liposarcoma from benign lipoma [11]. *MDM2* and *CDK4* are located on chromosome 12q13-15, an area that is amplified in well-differentiated and dedifferentiated liposarcoma [11]. Immunohistochemistry for MDM2 and CDK4, when strong and diffuse, can be used as a surrogate for FISH with reasonable diagnostic efficacy [12].

## 4. Conclusions

Although rare, liposarcoma of the head and neck is important to recognize. It can be misdiagnosed due to infrequency and subtle histological findings. The prognosis of liposarcoma in the hypopharynx is better than other sites, possibly due to earlier detection.

## Abbreviation

FNCLCC: Fédération Nationale des Centres de Lutte Contre Le Cancer
CT: Computerized tomography
PET CT: Positron emission tomography computerized tomography
BCL2: B-cell lymphoma 2
CD: Cluster of differentiation
CK OSCAR: Cytokeratin OSCAR
SOX10: SRY-related HMG-box 10
SMA: Smooth muscle actin
CDK4: Cyclin-dependent kinase 4
ERG: Erythroblast transformation-specific-related gene
FISH: Fluorescence in situ hybridization
MDM2: Mouse double minute 2 homolog
LS: Liposarcoma
ALT: Alanine aminotransferase
WDLS: Well-differentiated liposarcoma
DDLS: Dedifferentiated liposarcoma.
